# Is breathing frequency a potential means for monitoring exercise intensity in people with atrial fibrillation and coronary heart disease when heart rate is mitigated?

**DOI:** 10.1007/s00421-024-05487-2

**Published:** 2024-05-04

**Authors:** John P. Buckley, Tasuku Terada, Anna Lion, Jennifer L. Reed

**Affiliations:** 1 School of Allied Health Professions, Keele University, Staffordshire, ST5 5BG UK; 2 University of Ottawa Heart Institute, Ottawa, ON Canada; 3 School of Epidemiology and Public Health, Faculty of Medicine, University of Ottawa, Ottawa, ON Canada; 4 Physiology, Pharmacology and Neuroscience, School of Life Sciences, University of Nottingham, Nottingham, UK; 5 Rehabilitation Technologies Network+, Faculty of Engineering, University of Nottingham, Nottingham, UK

**Keywords:** Breathing rate, Heart rate, Anaerobic threshold, Cardiac rehabilitation

## Abstract

**Purpose:**

Moderate-intensity aerobic exercise is safe and beneficial in atrial fibrillation (AF) and coronary heart disease (CHD). Irregular or rapid heart rates (HR) in AF and other heart conditions create a challenge to using HR to monitor exercise intensity. The purpose of this study was to assess the potential of breathing frequency (BF) to monitor exercise intensity in people with AF and CHD without AF.

**Methods:**

This observational study included 30 AF participants (19 Male, 70.7 ± 8.7 yrs) and 67 non-AF CHD participants (38 Male, 56.9 ± 11.4 yrs). All performed an incremental maximal exercise test with pulmonary gas exchange.

**Results:**

Peak aerobic power in AF (${\dot{\text{V}}}$O_2_peak; 17.8 ± 5.0 ml.kg^−1^.min^−1^) was lower than in CHD (26.7 ml.kg^−1^.min^−1^) (*p* < .001). BF responses in AF and CHD were similar (BF peak: AF 34.6 ± 5.4 and CHD 36.5 ± 5.0 breaths.min^−1^; *p* = .106); at the 1st ventilatory threshold (BF@VT-1: AF 23.2 ± 4.6; CHD 22.4 ± 4.6 breaths.min^−1^; *p* = .240). %${\dot{\text{V}}}$O_2_peak at VT-1 were similar in AF and CHD (AF: 59%; CHD: 57%; *p* = .656).

**Conclusion:**

With the use of wearable technologies on the rise, that now include BF, this first study provides an encouraging potential for BF to be used in AF and CHD. As the supporting data are based on incremental ramp protocol results, further research is required to assess BF validity to manage exercise intensity during longer bouts of exercise.

## Introduction

The prevalence of atrial fibrillation (AF) is linked to a variety of factors, including: an ageing population, smoking, obesity, hypertension, or as a secondary condition to other cardiac diseases/dysfunction (Morseth et al. 2021; Morillo et al. 2017). As a secondary condition, AF can occur in up to 30% of people with either angina, heart failure, cardiac myopathies, myocardial infarction, after arterial or valvular heart surgery, or those who have engaged in high volumes of sport and exercise training for many years (Morin et al. 2016; Newman et al. 2021; Andersen et al. 2013). Appropriately guided exercise for those with AF has shown similar physiological and psychosocial health benefits to those with coronary heart disease (CHD) or heart failure (Reed et al. 2013, 2018; Smart et al. 2018).

With the known effects of AF on heart rate (HR) and breathing (Lip et al. 2016), the use of traditional cardiac rehabilitation guidance on setting and monitoring exercise intensity either using heart rate or ventilatory responses (Etiwy et al. 2019) is seemingly challenged. Normally, following an exercise test, target exercise training intensities are set to a relative percentage (%) of peak or maximum heart rate (%HRmax or % heart rate reserve), % maximal oxygen uptake (%${\dot{\text{V}}}$O_2_max), or % maximal aerobic work rate (Iannetta et al. 2020; Anselmi et al. 2021; Gati et al. 2020). More precise techniques recommend setting target HRs or work-rates which correspond to the first and second ventilatory thresholds (VT-1, VT-2, respectively), depending on whether continuous or interval training approaches are used (Pattyn et al. 2018). When the reliable and valid use of HR is challenged (e.g. in people with dysrhythmias like AF), could breathing frequency be a suitable alternative to represent a given %HRmax/reserve, %${\dot{\text{V}}}$O_2_max or VT-1or VT-2?

Currently, in the absence of using HR to monitor intensity, pragmatic recommendations for self-monitored exercise intensity are typically left to the use of ratings of perceived exertion/breathing effort/breathlessness (Borg 1998; Spruit et al. 2013; Buckley and Thow 2006; Buckley et al. 2009). With perceived breathing effort or breathlessness being a staple tool of cardiopulmonary rehabilitation (Chen et al. 2002), it seems intuitively perplexing that for the past four decades there has been no parallel technology pursuit to HR to similarly monitor breathing effort objectively. Only in the past 5 years has there been some development of commercial devices to measure exercise breathing effort (including frequency) (Nicolo et al. 2017, 2020). However, in those devices that report breathing frequency (BF), the measures are usually derived from algorithms using HR/ECG variability (Massaroni et al. 2019 a, b). A case for ECG-derived measures of BF in AF has been put forward but only during simulated rest signals (not using actual patients) and with limited applications to sleep apnoea (Kontaxis et al. 2020). A case against the use of ECG or HR variability-derived BF in AF has been reported by Platisa et al. (2016), who demonstrated an uncoupling of heart rate and BF in AF. BF derived from personal HR monitors will likely use either single-lead ECG frequency spectrums, HR variability algorithms (Chung et al. 2021) or second level derivations based on pulse-plethysmography from wrist- or arm-band wearables (Lazaro et al. 2018). Encouragingly, there are some emerging technologies that are reporting BF directly from either chest wall motion sensors or expired mouth-air sensors, but these have not yet reached the wider public market, let alone being validated for use with patient populations.

**Fig. 1:**
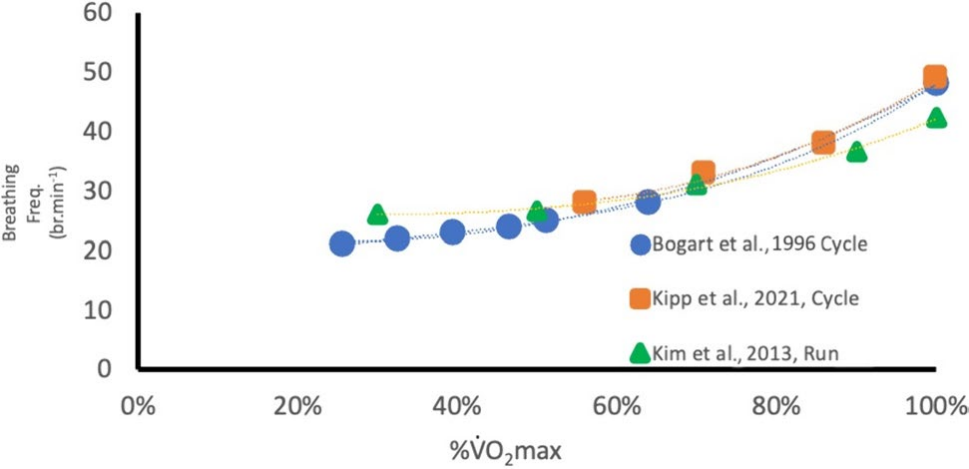
Re-adapted data from three studies in athletic populations to illustrate the potential value of the association between breathing frequency (br^.^min^−1^) and relative aerobic power (%${\dot{{V}}}$O_2_max)

In the optimistic anticipation that technology will soon evolve towards more practical, valid and affordable means of measuring BF for use in exercise in healthy and clinical populations (Nicolo et al. 2020), we sought to assess the underpinning physiological rationale/evidence for the value of BF at least in healthy populations. In this assessment, limited data were found on the reporting of BF during incremental exercise at submaximal and up to maximal efforts that could be adapted to our aims. There was one study during submaximal and three relevant studies during incremental maximal effort in healthy athletic populations using cardiopulmonary exercise tests (CPET) (Bogaard et al. 1996; Kim et al. 2013; Kipp et al. 1985; Cummin et al. 1986). From the maximal test data, the “mean” data (Fig. 1) were plotted to illustrate potential relationships between relative aerobic power (%${\dot{\text{V}}}$O_2_max) and BF to assess its possible utility in a similar way to HR. Across these three studies there appeared to be a consistent relationship of BF with %${\dot{\text{V}}}$O_2_max with a between-studies variation of ~ 5–8 br.min^−1^ at any given %${\dot{\text{V}}}$O_2_max The expected curvilinear nature of the BF kinetics (Balady et al. 2010) is clearly visible in Fig. 1, raising the hypothesis that similar to athletic populations, BF could also be used to represent a reliable exercise response curve in AF for factors such as the identification of ventilatory thresholds (Carey et al. 2005). The assumptions of such a rationale are that VT-1 denotes the lower end of moderate-intensity aerobic endurance exercise and VT-2, also known as the respiratory compensation threshold, represents vigorous- to high-intensity aerobic endurance exercise (Balady et al. 2010; Mezzani et al. 2013).

Assuming the same features of CPET demonstrated in Fig. 1 can be applied in cardiopulmonary diseased populations, we therefore sought in this current study to evaluate the relationship between BF and the typically recommended cardiac rehabilitation “moderate” exercise intensity parameters of HR (where possible), %${\dot{\text{V}}}$O_2_peak and VT-1 in adults with persistent and permanent AF and non-AF CHD (the comparator group). Our ultimate goal, dependent on emerging technology, is to explore the use of BF instead of HR to represent submaximal aerobic exercise intensity across a number of cardiac conditions, in both testing and training scenarios, (e.g. AF, heart failure, bradycardia, tachycardia, those on chronotropic medications or medications that have chronotropic side effects).

## Methods

### Ethical approval, equity, diversity, inclusion

The BF and related physiological measures of participants with AF and CHD (without AF) collected in previously published work (Reed et al. 2019, 2022) were analysed. These studies were designed with *patient and public involvement*. Additional ethical approval was obtained from the Ottawa Health Science Network Research Ethics Board to use and share these data sets for the purposes of this new analysis and with new collaborators. The study was performed in accordance with the ethical standards as outlined in the 1964 Declaration of Helsinki and its later amendments or comparable ethical standards.

All of the publishers’ criteria for patient equity, diversity and inclusion are standard and requisite practice within the normal referral/recruitment to the patient services where the research occurred. This has been previously published (Reed et al. 2019, 2022), and it must be appreciated that recruiting eligible females and ethnic minorities to cardiac rehabilitation is currently a widely reported challenge (Mehra et al. 2020).

### Participants

The detailed recruitment methodologies for both AF and CHD have been described previously (Reed et al. 2022, 2019). The inclusion criteria for the AF patients included: (1) documented persistent or permanent AF as confirmed by a physician; (2) rate-controlled with a resting HR of ≤ 100 bpm; (3) ability to perform CPET to exhaustion; and, (4) age ≥ 40 years. Patients were excluded if they already participated in routine exercise training more than twice a week, had a diagnosis of chronic obstructive pulmonary disease, severe mitral or aortic stenosis, or hypertrophic cardiomyopathy with obstruction. Details of the recruitment process, inclusion criteria and study protocol for the CHD participant data were published previously (Reed et al. 2019).

### Cardiorespiratory exercise testing and data analyses

Cardiorespiratory fitness was measured in both AF and CHD participants using symptom-limited ramp protocols with respiratory gas exchange measured by CPET. AF participants were assessed using a portable CPET (Carefusion Oxycon Mobile) while exercising on an electronically braked cycle ergometer (COSMED, USA) with the details previously published (Reed et al. 2022). The initial cycle ergometer work rate was set at 40 Watts for the first 2 min and increased by 20W every minute thereafter. CHD participants were assessed on a treadmill with gas exchange measured by a lab-based metabolic cart (Sensormedics Vmax, Yorba Linda, CA, United States), with testing details described previously (Reed et al. 2019). For both AF and CHD participants, gas exchange and BF were monitored continuously, and the highest rate of oxygen uptake achieved during the last minute of the CPET represented peak aerobic power (peak ${\dot{\text{V}}}$O_2_ in ml^.^kg^−1.^min^−1^). For both AF and CHD participants, VT-1 and VT-2 were determined by plotting ${\dot{\text{V}}}$CO2, ventilation (${\dot{\text{V}}}$E), ${\dot{\text{V}}}$E/${\dot{\text{V}}}$O2, and ${\dot{\text{V}}}$E/${\dot{\text{V}}}$CO2 against ${\dot{\text{V}}}$O2. VT-1 and VT-2 would be used as the target moderate exercise intensity reference point to assess the utility of a corresponding BF (Mezzani et al. 2013; Arena and Sietsema 2011).

Differences of within- or between-participant group data (e.g., AF vs. CHD) were assessed using *t* tests, and associations between measures were assessed with a Pearson correlation coefficient (*r*). Slope estimates of BF relative to exercise intensity for linear or curvilinear functions were determined by exponential or polynomial growth models using MATLAB (version R2022a), as exampled on existing data in Fig. 1. The level of statistical significance was set at *p* < 0.05. Data can be made available upon reasonable request.

## Results

**Fig. 2:**
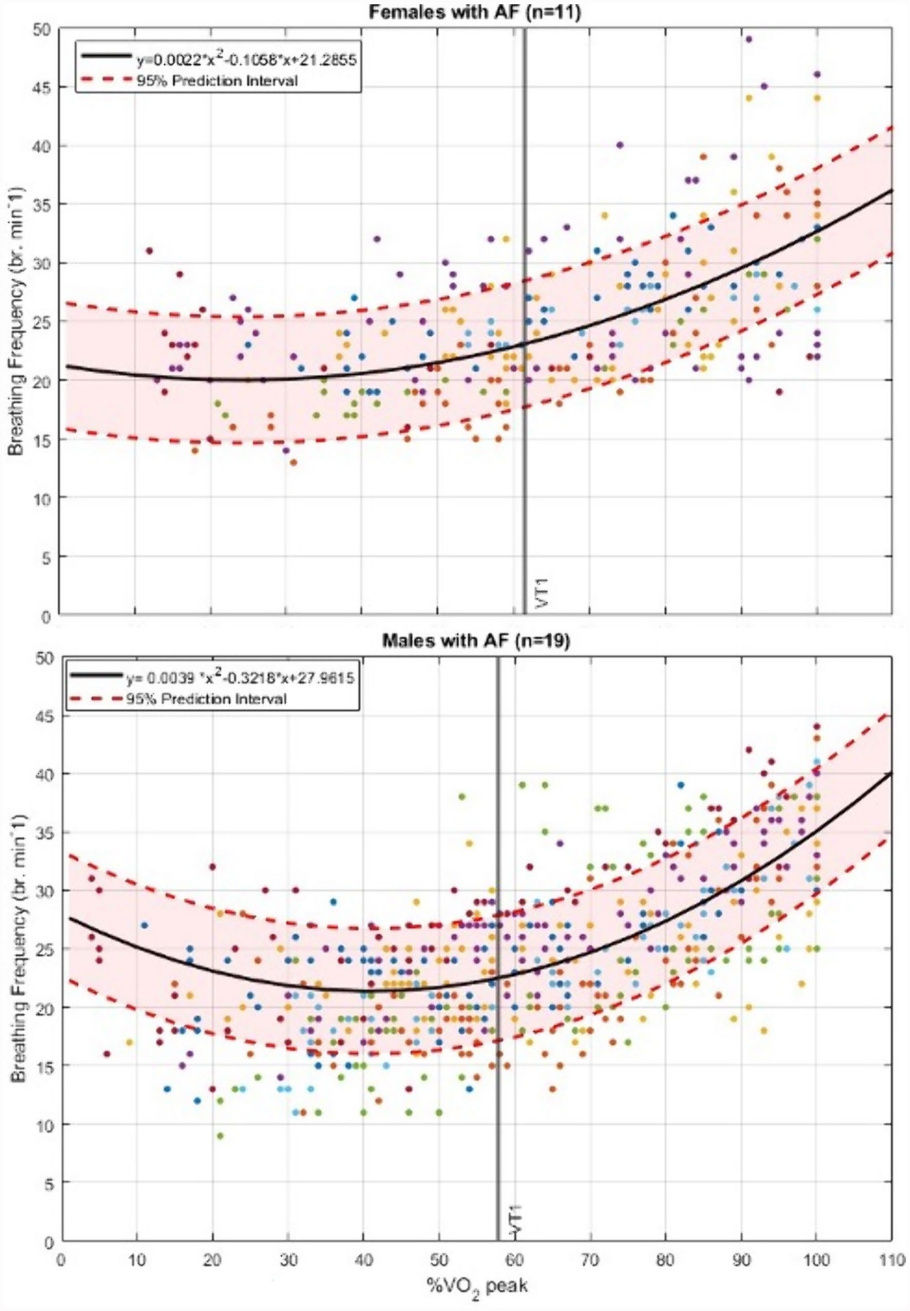
Individual atrial fibrillation patient breathing frequency responses, as a function of %*V*O_2_peak during incremental exercise in 19 males (top graph) and 11 females (bottom graph). Note the curvilinear nature of the plots with VT-1 (1.^st^ ventilatory threshold) occurring at near 60%*V*O_2_ peak in both males and females; y = the group mean second-order polynomial regression curve for predicting BF from %*V*O_2_peak (see Table 1)

**Fig. 3:**
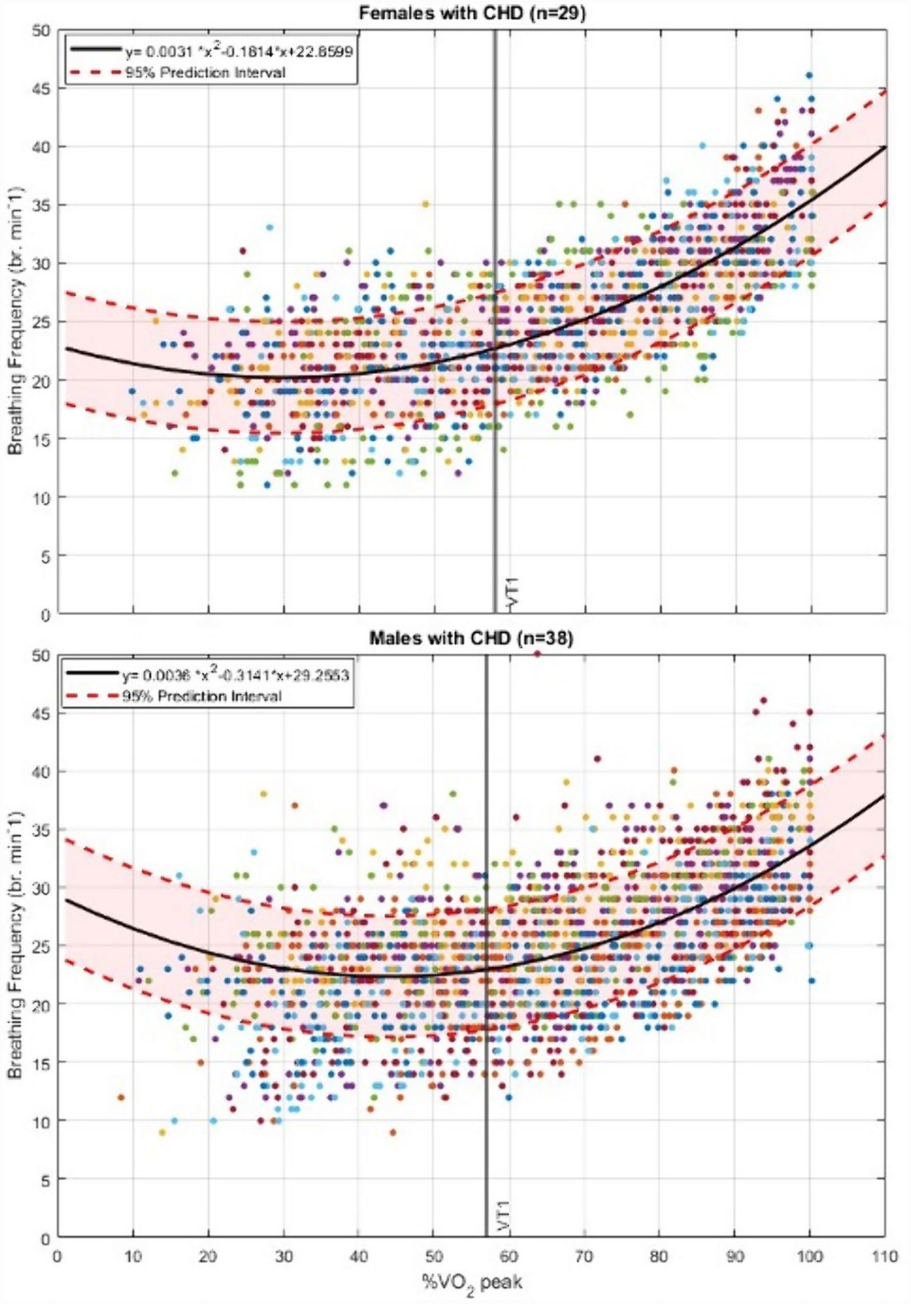
Individual coronary heart disease patient breathing frequency responses, as a function of %*V*O_2_peak during incremental exercise in 37 males (top graph) and 28 females (bottom graph). Note the curvilinear nature of the plots with VT-1 (1^st^ ventilatory threshold) occurring at near 60%*V*O_2_ peak in both males and females; y = the group mean second-order-polynomial regression curve for predicting BF from %*V*O_2_peak (see Table 2)

The key health measures from 97 cardiac rehabilitation participants included CPET responses from 30 AF participants (19 males, 3 with CHD) and 67 CHD participants (38 males) (Tables 1 and 2). In keeping with contemporary research and publishing recommendations (Clayton and Tannenbaum 2016), we have provided the separate male and female data to assist any future studies or interested research groups who may wish to use/compare our sex-specific data. Median and mean exponential and polynomial growth curves were modelled through the BF data as a function of % ${\dot{\text{V}}}$O_2_peak. As the 2nd order polynomial curve matched well with the individual ventilatory equivalents (${\dot{\text{V}}}$E/ ${\dot{\text{V}}}$O_2_, ${\dot{\text{V}}}$E/ ${\dot{\text{V}}}$CO_2_) linked to VT-1, it was chosen as the preferred regression model as illustrated in Figs. 2 and 3. As there was little observable difference in the mean versus median growth curves, the mean curve was chosen to represent the group responses for the analyses.

**Table 1:** Health and cardiopulmonary exercise testing measures in males and females with persistent and permanent atrial fibrillation

	Male *n* = 19	SD	Female *n* = 11	SD	Pooled M & F	SD
Age (years)	69.8	8.8	72.3	8.7	70.7	8.7
Height (cm)	177.4	4.7	167.7	10.4	173.8	8.6
Mass (kg)	97.4	20.5	89.6	25.8	94.6	22.5
BMI (kg/m^2^)	31.0	6.7	31.8	8.6	31.3	7.3
${\dot{\text{V}}}$O_2_peak (ml^.^kg^−1.^min^−1^)	19.0	5.0	15.7	4.5	17.8	5.0
RER peak	1.2	0.1	1.2	0.1	1.2	0.1
BF peak (br^.^min^−1^)	35.5	3.9	33.2	7.3	34.6	5.4
BF @ VT (br^.^min^−1^)	23.2	4.8	23.1	4.4	23.2	4.6
%BF peak @VT-1	65.4	11.1	71.5	15.1	67.6	12.8
%${\dot{\text{V}}}$O_2_peak @VT-1	57.8	13.5	60.1	10.9	58.6	12.5
${\dot{\text{V}}}$E/${\dot{\text{V}}}$O_2_ @VT-1	32.8	4.8	31.3	4.5	32.3	4.6
${\dot{\text{V}}}$E/${\dot{\text{V}}}$CO_2_ @VT-1	33.7	5.0	34.1	4.5	33.9	4.8

${\dot{\text{V}}}$*O*_*2*_ rate of oxygen uptake, *RER* respiratory exchange ratio, *BF* breathing frequency (br^.^min^−1^), *BMI *body mass index, *VT* ventilatory threshold, *VT-1* the first ventilatory threshold; ${\dot{\text{V}}}$*E/*${\dot{\text{V}}}$*O*_*2*_* and *${\dot{\text{V}}}$*E/*${\dot{\text{V}}}$*CO*_*2*_ ventilatory equivalents for rates of oxygen uptake and carbon dioxide expiration, respectively

**Table 2:** Health and cardiopulmonary exercise testing measures in males and females with coronary heart disease

	Male *n* = 38	SD	Female *n* = 29	SD	Pooled M & F	SD
Age (years)	57.7	9.6	55.9	13.4	56.9	11.4
Height (cm)	174.8	6.9	161.3	7.6	168.9	9.8
Mass (kg)	86.7	15.0	72.5	16.3	80.5	17.0
BMI (kg/m^2^)	28.4	4.6	27.9	6.4	28.2	5.4
${\dot{\text{V}}}$O_2_peak (ml^.^kg^−1.^min^−1^)	26.9	6.0	26.5	8.0	26.7	6.9
RER peak	1.1	0.1	1.1	0.1	1.1	0.1
BF peak (br^.^min^−1^)	35.4	4.8	37.9	5.0	36.5	5.0
BF @ VT (br^.^min^−1^)	22.3	4.9	22.5	4.4	22.4	4.6
%BF peak @VT-1	63.1	11.2	59.8	11.5	61.7	11.3
%${\dot{\text{V}}}$O_2_peak @VT-1	57	8.8	58.0	10.4	57.4	9.5
${\dot{\text{V}}}$E/${\dot{\text{V}}}$O_2_ @VT-1	25.3	4.2	26.2	5.5	25.7	4.8
${\dot{\text{V}}}$E/${\dot{\text{V}}}$CO_2_ @VT-1	31.6	4.5	33.5	4.7	32.4	4.7
%HRpeak @ VT-1	73	6	70	7	72	7

${\dot{\text{V}}}$*O*_*2*_ rate of oxygen uptake, *RER* respiratory exchange ratio, *BF* breathing frequency (br^.^min^−1^), *BMI* body mass indx, *VT* ventilatory threshold, *VT-1* the first ventilatory threshold, ${\dot{\text{V}}}$*E/*${\dot{\text{V}}}$*O*_*2*_* and *${\dot{\text{V}}}$*E/*${\dot{\text{V}}}$*CO*_*2*_ ventilatory equivalents for rates of oxygen uptake and carbon dioxide expiration, respectively; *HRpeak* peak heart rate

### Atrial fibrillation participants

Of the 30 AF participants, 13 had persistent AF and 17 had permanent AF. The group BMI (> 31) is categorized as obese, with one third of participants having a BMI > 35 kg^.^m^−2^. The individual patient BF responses, as a function of relative aerobic exercise intensity (%${\dot{\text{V}}}$O_2_peak), are illustrated in Fig. 2. The group mean BF at VT-1 occurred at 59% ${\dot{\text{V}}}$O_2_peak and was ~ 23 br·min^−1^ (br·min^−1^) with inter-individual variations (1 SD) of 4.5 br·min^−1^ (Table 1).

Whilst the mean age of the AF cohort was nearly 70 years, it is of note that two of the female participants were 83 and 90 years, with ${\dot{\text{V}}}$O_2_peak values of 14.5 and 17.0 ml^.^kg^−1.^min^−1^, respectively. For the whole group, peak BF was weakly negatively but significantly correlated with age (*r* = − 0.42, p = 0.02). For both males and females peak BF was very weakly correlated with ${\dot{\text{V}}}$O_2_peak (*r* < 0.15, *p* = 0.46). In thoroughly evaluating criteria for ${\dot{\text{V}}}$O_2_max vs. ${\dot{\text{V}}}$O_2_peak, 25 of 30 participants attained respiratory exchange ratios > 1.10 (40). At VT-1, the group’s mean (SD) for %BF peak was 67.6% (12.8%) and %${\dot{\text{V}}}$O_2_peak was 58.6% (12.5%) (Table 1, Fig. 2). Observation of HR responses in the AF group demonstrated random fluctuations, as expected in most participants and thus rendered such data not valid for reporting and analysing.

### Coronary heart disease participants

The characteristics of patients with CHD are summarised in Table 2. The individual patient BF responses, as a function of relative aerobic exercise intensity (%${\dot{\text{V}}}$O_2_peak), are illustrated in Fig. 3. The group mean BF at VT-1 occurred at 57%${\dot{\text{V}}}$O_2_peak and was ~ 22 br·min^−1^, with inter-individual variations (1 SD) of 4.6 br·min^−1^ (Table 2). For the CHD group, peak BF was not significantly correlated with age (*r* = − 0.067., *p* = 0.592). Peak BF was correlated with ${\dot{\text{V}}}$O_2_peak in females (*r* = 0.375 *p* = 0.049) but not in males (*r* = 0.163, *p* = 0.328). In evaluating the criteria for ${\dot{\text{V}}}$O_2_max vs. ${\dot{\text{V}}}$O_2_peak, 36 of 67 participants achieved respiratory exchange ratios > 1.10 (40). At VT-1, the group’s mean (SD) for %BF peak was 61.7 ± 11.3% (Table 2, Fig. 3). Mean %HRpeak in the CHD group at VT-1 for males and females were 73 ± 6% and 70 ± 7%, respectively, with a pooled mean of 72 ± 7% (Tables 2 and 3).

**Table 3:** Comparison of health and cardiopulmonary exercise testing measures between AF and CHD participants (pooled male and female data)

	AF (*n* = 30)	CHD (*n* = 67)	*P* values	95% CI
Age years	70.7 (8.7)	56.9 (11.4)	** < 0.001**	**− 18.0, − 9.6**
BMI (kg.m^2^)	31.3 (7.3)	28.2 (5.4)	**0.021**	−** 5.8, **−** 4.9**
${\dot{\text{V}}}$O_2_peak (ml^.^kg^−1.^min^−1^)	17.8 (5.0)	26.7 (6.9)	** < 0.001**	**6.1, 11.7**
BF peak (br^.^min^−1^)	34.6 (5.4)	36.5 (5.0)	0.106	− 0.4, 4.1
BF @ VT-1 (br^.^min^−1^)	23.2 (4.6)	22.4 (4.6)	0.240	− 3.3, 0.8
%BF peak @ VT-1	67.6 (12.8)	61.7 (11.3)	**0.007**	**− 12.5, − 2.1**
%${\dot{\text{V}}}$O_2_peak @ VT-1	58.6 (12.8)	57.4 (9.5)	0.656	-17.9, 10.0
%HRpeak @ VT-1	–	72 (7)	–	–

${\dot{\text{V}}}$*O*_*2*_ rate of oxygen uptake, *RER* respiratory exchange ratio, *BF* breathing frequency (br^.^min^−1^), *BMI* body mass index, *VT* ventilatory threshold, *VT-1* the first ventilatory threshold; *HRpeak* peak heart rate

### Comparison of AF and CHD participants

Table 3 summarises a comparison of the health and exercise testing response differences between the AF and CHD participants. Compared to the CHD participants, those with AF had the following significant differences: 13.8 years older; a greater BMI of 3.1 kg/m^2^; and 33% lower ${\dot{\text{V}}}$O_2_peak (− 8.9 ml^.^kg^−1.^min^−1^).

AF and CHD participants had very similar BF and %${\dot{\text{V}}}$O_2_peak values at VT-1, but the AF participants were exercising at a significantly greater %BF peak (67.7% vs 61.7%, *p* = 0.007).

## Discussion

To the best of our knowledge, this is the first study to assess BF during incremental peak exercise in adults with AF and CHD (without AF). Our goal was to examine a potential alternative pragmatic means to the traditional use of HR for monitoring exercise intensity in a group of people where measuring HR is known to be problematic. Our main finding was that in individuals with AF and CHD (all aged > 55 years), exercising at a BF of 22 to 25 br·min^−1^ represented a safe and beneficially recommended moderate exercise intensity as defined by either %HRpeak, %${\dot{\text{V}}}$O_2_peak or VT-1 (Anselmi et al. 2021; Gati et al. 2020; Bosomworth 2015; Mozaffarian et al. 2008; Milani et al. 2023). Future pragmatic research around setting an efficacious target BF during steady state exercise (e.g. 22 to 25 br·min^−1^) is needed in respect of our currently reported 20% SD (4 to 5 br·min^−1^) at VT-1. However, such a 20% range is similar to that reported for %HRpeaks found at VT-1 (Milani et al. 2023). Whilst we noted in Fig. 1 that in athletes the BF showed promising associations with the ventilatory or anaerobic threshold (Carey et al. 2005), those reports did not specifically analyse or provide any related conclusions linked to BF. If a comparison can be made with Fig. 1, it relates to the %BFpeak range we reported (Figs. 2 and 3) at VT-1, which aligned to 57–59%${\dot{\text{V}}}$O_2_peak (Table 3). When comparing BF in the athletes of Fig. 1 at 57–59% ${\dot{\text{V}}}$O_2_peak, they had BF values of 27–29 br·min^−1^ (55–65% of their BFpeak). This is similar to our CHD and AF participants at this intensity, with %BF peaks of 61% and 67%, respectively. It does, however, need to be acknowledged that for Fig. 1 we estimated the athlete BF response curves from a single group-mean data point versus the mean of the individual participant’s response curves in our data. We are unaware of any other similar data from which to compare our results for healthy or disease-specific populations.

### Breathing frequency responses compared to historically accepted theories of cardiopulmonary pulmonary dynamics and exercise prescription

In comparing our BF response curves (Figs. 2 and 3), as a function of relative exercise intensity (%${\dot{\text{V}}}$O_2_peak), with those from the limited evidence-base of younger and trained participants (Fig. 1), we too found a clear curvilinear line. Figure 1, however, only represents responses for intensities > 25% ${\dot{\text{V}}}$O_2_peak, where ours are taken from a starting point of rest and moving through initial lower exercise stages at ≤ 15% ${\dot{\text{V}}}$O_2_peak. Intuitively one might expect to see a simple positively accelerating BF curve similar to that of minute ventilation or ${\dot{\text{V}}}$CO_2_ during incremental exercise (Beaver et al. 1985; Diamond et al. 1977). However, our BF responses more closely resembled a U-shaped ventilatory-equivalent curve for oxygen uptake (${\dot{\text{V}}}$E/${\dot{\text{V}}}$O_2_) during incremental exercise from rest up to maximum effort (Diamond et al. 1977). Even with the onset or acute increments of light-moderate intensities of exercise, BF among other ventilatory factors have previously shown to respond with an abrupt “jump” in activity within less than two-minutes (Cummin et al. 1986; Diamond et al. 1977). Specific to breathing frequency, Cummin et al. (1986) reported, during four incremental submaximal bouts of cycling (18 min per bout), an acute “jump” in BF of 4 to 7 br·min^−1^ (25–50%) in the first two-minutes of each bout; but within 3 to 5 min it then dropped back down by 3 to 4 br·min^−1^ to remain at a steady state for the remainder of each 18 min bout. Interestingly this downward drift in BF during prolonged steady state exercise was at its lowest value at the end of the 18 min. This downward drift in BF is opposite to the widely known upward drift of HR that typically occurs over this same period (Souissi et al. 2021).

### Translating ramp protocol exercise test responses into longer bouts of subsequent exercise training

As our data in Figs. 2 and 3 are based on ramp protocols with one-minute stages, the above evidence by Cummin et al. does help to provide a clearer rationale to why the BFs at the low-moderate intensities were higher than at VT-1, and then increased to maximum producing a U-shaped curve. This likely raises the most important pragmatic application challenge of this current study, on the validity of taking a BF collected from a ramped-incremental exercise test with short duration stages, and translating it into a subsequent target BF for more prolonged exercising training bouts. Future research is thus required to develop such differentiated models of testing vs training BF response targets when considering it as an alternative monitoring tool to HR and ratings of perceived exertion/breathlessness typically used in rehabilitative exercise.

### ***BMI and ***${\dot{{V}}}$*O*_***2***_***peak***

The mean BMI of the AF group was 31 kg·m^−2^, and one third of these patients had a BMI > 35 kg·m^−2^, which is expected as one of the key associated risk factors for AF (Morseth et al. 2021; Morillo et al. 2017). The ${\dot{\text{V}}}$O_2_peak of the AF males (19.0 ml^.^kg^−1.^min^−1^) and females (15.7 ml^.^kg^−1.^min^−1^) would rank these participants at the lower percentiles for fitness; specifically in the 10th and 5th percentiles, respectively, for age-matched Canadians aged ~ 70 years (Lewthwaite et al. 2020; Hoffmann et al. 2019). Whilst the 33% lower ${\dot{\text{V}}}$O_2_peak in the AF compared with CHD participants agrees with a number of existing reports (Reed et al. 2018; Elliott et al. 2021; Atwood et al. 2007; Ueshima et al. 1993), there are two strong confounding contributing factors that warrant mentioning: i. the AF participants were on average 14 years older and ii. they performed their exercise tests on a cycle ergometer (Astrand et al. 2003).

### Peak breathing frequency

The peak BF of the AF males and females (35 and 33 br·min^−1^, respectively) were similar to the CHD participants (35 and 37 br·min^−1^, respectively) and age-matched non-AF populations (Lewthwaite et al. 2020; Roman et al. 2016). However, compared to younger athletic populations (Bogaard et al. 1996; Kim et al. 2013; Kipp et al. 1985), a submaximal BF of 22–25 would likely be associated with a lower relative exercise intensity (40–50% ${\dot{\text{V}}}$*O*_*2*_*max*), given that in young healthy populations the BF peak has been reported to be 40 to 50 br·min^−1^. Consistent with these higher BF values in younger populations, we demonstrated a weak but significant negative correlation between age and breathing frequency. However, our group was limited to a mean age range of 15 years, where we may have lacked a wide enough spread of data at the bottom and top of the age range to determine a more meaningful correlation. The question of whether our participants reached a true maximal BF and ${\dot{\text{V}}}$O_2_ is partially answered as “yes” for AF where 25 of 30 AF participants attained one of the key “maximal” criteria with respiratory exchange ratios of > 1.10 (Balady et al. 2010; Lewthwaite et al. 2020). However, in the CHD participants 47% of them did not attain a peak RER > 1.10, which may indicate they had even a greater ${\dot{\text{V}}}$O_2_peak and BF peak, compared to AF participants, than what we have reported.

### Breathing frequency and key cardio-ventilatory response thresholds

As noted above, a BF range of 22 to 25 br·min^−1^ coincided with VT-1 and represented 57–60% ${\dot{\text{V}}}$O_2_peak in both AF and CHD participants, which is within the range for healthy untrained individuals (45–65% ${\dot{\text{V}}}$O_2_max) (Balady et al. 2010). For the CHD patients, the 70–73% HRpeak occurring in this BF range also provides some confidence in relation to meeting current cardiac rehabilitation guidelines (Gati et al. 2020). However, an area that does raise questions of AF and CHD is the altered normal exercise respiratory dynamics found in the ventilatory equivalents (${\dot{\text{V}}}$E/${\dot{\text{V}}}$O_2_ and ${\dot{\text{V}}}$E/${\dot{\text{V}}}$*CO*_*2*_) (Mezzani et al. 2013). In AF both ${\dot{\text{V}}}$E/${\dot{\text{V}}}$O_2_ and ${\dot{\text{V}}}$E/${\dot{\text{V}}}$CO_2_ had respective ratios > 31 and 33 at VT-1 (Table 2) and in CHD ${\dot{\text{V}}}$E/${\dot{\text{V}}}$CO_2_ was > 32. These values do border on clinically significant levels for impaired cardiac or pulmonary function/disease (Balady et al. 2010; Mezzani et al. 2013; Nusair 2017). Furthermore, recent evidence has confirmed such declines in ventilatory efficiency being a specific clinical feature in AF (Elliott et al. 2021).

### Conclusions, clinical implications and limitations

This study has laid down some encouraging groundwork for a “potential” new way of monitoring exercise intensity in rehabilitation populations when normal assumptions about exercise HR dynamics become mitigated (e.g. heart failure, pulmonary disease, cancer). As innovative technologies continue to emerge, practitioners and patients could potentially have another means, other than HR, to assess physiological strain and aid in patient self-monitoring of exercise intensity. At this point, it is important to respect that the pragmatics of using BF during actual exercise training sessions is yet to be fully assessed. Further work is thus required to evaluate how the relationship between BF and other more traditional cardiopulmonary responses occur during extended duration constant load moderate-intensity or higher-intensity interval training bouts.

## Data Availability

The datasets generated and analyzed during the current study are available from the corresponding author on reasonable request.

## Abbreviations

AF: Atrial fibrillation
BMI: Body mass index
BF: Breathing frequency
bpm: Beats per minute of heart rate
br.min^−^^1^: Breaths per minute
CHD: Coronary heart disease
CPET: Cardiopulmonary exercise test
ECG: Electrocardiogram
HR: Heart hate
%HRmax: Percentage of maximal heart rate
%HRpeak: Percentage of peak heart rate
HRR: Heart rate reserve
%HRR: Percentage of heart rate reserve
p: Level of statistical significance (alpha)
*r*: Correlation coefficient
SD: Standard deviation
$${\dot{\text{V}}}$$ E: Minute ventilation
${\dot{\text{V}}}$ E/ ${\dot{\text{V}}}$CO_2_: Ventilatory equivalent for rate of carbon dioxide production
${\dot{\text{V}}}$ E/ ${\dot{\text{V}}}$O_2_: Ventilatory equivalent for rate of oxygen uptake
${\dot{\text{V}}}$ CO_2_: Rate of carbon dioxide production
${\dot{\text{V}}}$ O_2_: Rate of oxygen uptake
${\dot{\text{V}}}$ O_2_ max: Rate of maximal oxygen uptake or maximal aerobic power
${\dot{\text{V}}}$ O_2_ peak: Rate of peak oxygen uptake or peak aerobic power
VT-1: 1St ventilatory threshold
